# The Book World of Medicine and Science

**Published:** 1898-08-13

**Authors:** 


### The Book World of Medicine and Science.

Lectures on Medicine to Nurses. By Herbert E. Cuff, M.D. Second edition. Pp. 271. Price 3s. 6d. (J. and A. Churchill. London. 1898.)

This volume, having reached its second edition, must be held to have achieved some measure of success. It is not a manual of nursing proper; it is an elementary text-book of medicine, written in the form of lectures and in very simple language. Necessarily there are many omissions; necessarily, too, many general rules and statements are put forward to which there are notable exceptions. We have every sympathy with those nurses who wish to learn something of the diseases they help to conquer. But we question if, on the whole, it be an advantage that they learn from books of this class. Possibly, if medical men were less ready to supply peptonised pathology and compressed diagnostics, nurses would appreciate more fully than they often do the importance of full knowledge and the enormous difficulties of the doctor's part in healing. These little books on medicine contain the truth, no doubt, but not the whole truth. It is very nice for nurses to see diagrams of "Nature's method of arresting hæmorrhage" (p. 202), but it would be far better were they told that when hæmoptysis occurs the patient is at once to be laid down, with the head low, and the worst lung undermost. We absolutely demur to the statement that hæmoptysis is very seldom directly fatal, and that the first thing to be done is to reassure the patient (p. 213). In every consumption hospital sudden death from hæmoptysis is a frequent occurrence, despite the fact that by the means just mentioned a prompt nurse can often check the flow and prevent asphyxia. On page 52 we note the usual confusion between the "quick" and "frequent" pulses, and on the next page the "volume" of the pulse is confused with the force of its "impulse." If nurses are to learn these things surely they should learn them accurately. Is it wise, too, to tell nurses, without reservation, that cirrhosis of the liver is a disease due to the excessive use of alcohol? (p. 229), or that peripheral neuritis is most commonly due to the prolonged use of alcohol? The last statement is a matter of opinion; the first is incorrect. If it were otherwise the wisdom of such remarks would still be doubtful. On the whole Dr. Cuff's book fulfils the purpose for which it was written, and no doubt the delivery of the lectures of which it is composed was attended with much profit to the auditors.

A Text-Book of Diseases of the Kidneys and Genito-Urinary Organs. By Professor P. Fürbringer. Translated by W. H. Gilbert, M.D. Vol. II. Pp. 370. Price 10s. 6d. (London: H. K. Lewis. 1898.)

This volume of Professor Fürbringer's work deals with calculous and suppurative affections of the kidney, diseases of the bladder and urethra, and the male generative organs. Its interest, then, despite the fact that operative measures are not discussed, is surgical rather than medical. The plan of the work is simple and direct, the language terse and well chosen, and although we may deplore the introduction of such barbarous words as "cysto-phthisis" and "cysto-spasmus," we find the translator's work on the whole well done. The frequent and copious references to authorities, the abundance and completeness of detail, the firm reliance on scientific methods of diagnosis, sufficiently attest its Teutonic origin; yet, although we note the omission of references to names such as Morris and Beck, Professor Fürbringer is far more appreciative of the work of British authors than are many of his countrymen. We notice that Professor Fürbringer declares he has never, in non-tuberculous cases, detected the Alvarez-Lavel smegma or pseudo-tubercle bacillus, and affirms that when bacilli of a tuberculous kind are met with in urine they have always proceeded from tuberculous foci. Many competent observers will not agree with these dicta. Another interesting point is that Professor Fürbringer inclines towards the view of Bümm that gonorrhœa is, in the female, frequently an endometritis and urethritis, and not a true vaginitis. The latter portion of the volume deals at some length with some of the most difficult and delicate problems that the surgeon is ever called upon to solve. Professor Fürbringer's treatment of these problems is courageous, often unconventional, and deserving of particular attention. Inasmuch as these topics are usually, by English authors, either shirked altogether or cursorily dismissed, Professor Fürbringer's handling of them cannot fail to be helpful to many practitioners. Although in this volume no very striking innovations in practice, or developments of theory, are brought forward, it will be acceptable to English surgeons from the wealth of allusion to Continental writers whose views, on many minor points, are in opposition to those of the British school.

The Building of Workhouses. By George H. Bibby, F.R.I.B.A. (Bradley T. Batsford, 94, High Holborn, W.C. Pp. 89, illus. 8. Price 2s. 1898.)

This little book of eighty-nine pages, with rather more than half as many pages of advertisements, appears to be one of a series intended apparently for the instruction of architects. We are not acquainted with the author's works on asylum construction, factory construction, or police stations and prisons, but if these productions are built on the same lines as the one now before us, we fear they will not be of very great service to the profession for whom they are intended. The book starts off with a sketchy and wholly superfluous dissertation on the housing of the poor in the middle ages and the time of Queen Elizabeth, winding up with an irrelevant quotation from "Oliver Twist," and passes thence by violent transition to the rules laid down at Whitehall for a "complete workhouse for a large union." The "points" issued by the Local Government Board form the skeleton to which the substance of the book is attached, but the practical information which is given to elucidate the rules laid down by the central authority is of the most meagre description. The only occasions on which the author seems to think it desirable to offer specific advice is when an opportunity occurs to advertise certain firms. For example, on page 29 a list of firms supplying apparatus for disinfection is given, but no attempt whatever is made to describe what process of disinfection is best to adopt. A few plans are given of parts of workhouses; they are carelessly drawn and of little practical use, and the block plans are of no value whatever. To a young architect seeking for information on the right principles of workhouse planning we can by no means recommend Mr. Bibby as a guide. Rather we would say make a careful study of the Local Government Board requirements together with the notes (not official but excellent nevertheless) in Knight's annotated edition, and you will have a clearer idea of what is wanted than can possibly be gathered from Mr. Bibby's book.

